# The impact of endovascular stents types on perioperative outcomes of ruptured abdominal aortic aneurysms: a single-center experience

**DOI:** 10.3389/fcvm.2024.1272389

**Published:** 2024-03-13

**Authors:** Huibo Ma, Xueyi Wang, Yangshuo Liu, Yongxin Li, Mingjin Guo

**Affiliations:** ^1^ Department of Vascular Surgery, Affiliated Hospital of Qingdao University, Qingdao, Shandong, China; ^2^ Department of Vascular Surgery, Rongcheng People's Hospital, Weihai, China

**Keywords:** rAAA, EVAR, endovascular stents, mortality, intraoperative complications

## Abstract

**Introduction:**

Ruptured abdominal aortic aneurysm (rAAA) represents a critically urgent vascular surgical condition, and endovascular aneurysm repair (EVAR) is a clinically effective treatment option. This study aims to investigate whether the type of intravascular graft used for ruptured abdominal aortic aneurysms has an impact on perioperative outcomes of EVAR.

**Methods:**

A retrospective analysis was conducted on patients who underwent EVAR for ruptured abdominal aortic aneurysm at a single medical center from 2019 to 2022. Patients who required simultaneous stent implantation in the renal arteries or visceral arteries, as well as those with ruptured aneurysms located in the para-renal, supra-renal, or thoracoabdominal regions, were excluded from the analysis. Additionally, patients who underwent open surgery during the initial procedure or converted to open repair were excluded. The primary endpoint was perioperative mortality rate. Other study outcomes included perioperative complications, reoperation rates, and length of hospital stay. Characteristics and corresponding outcomes of patients receiving different endovascular stent treatments were compared using SPSS software.

**Results:**

A total of 58 patients received treatment with two types of endovascular stents: Gore Excluder (*n* = 29) and Microport Hercules (*n* = 29). The number of other endografts was too small for statistical analysis. Compared to patients treated with Hercules, those treated with Excluder had a significantly increased likelihood of concomitant coronary atherosclerosis (*P* = 0.009) and potentially higher creatinine levels (*P* = 0.014). Additionally, Excluder was more commonly used in patients with shorter aneurysm necks (*P* < 0.001). There was a statistically significant difference in overall mortality between the two groups (Hercules 27.6%, Excluder 6.9%, *P* = 0.037). Furthermore, patients who received Excluder treatment had lower mortality rates in subgroups of non-alcohol users (*P* = 0.028), non-diabetic patients (*P* = 0.027), and patients with dispersed thrombosis at the proximal neck (*P* = 0.046). In the multivariate analysis, the type of stent used (OR 0.06, 95% CI 0.00–1.31) and the occurrence of intraoperative complications (OR 20.70, 95% CI 1.14–76.70) in patients with rAAA was identified as an independent risk factor for perioperative mortality.

**Conclusion:**

Our study suggests that the management of intraoperative complications may be a modifiable factor that can improve outcomes. Patients receiving Excluder treatment demonstrated better performance in EVAR for single-center rAAA patients compared to other endovascular stents, and this difference warrants further investigation.

## 1 Introduction

Rupture is the most severe complication of abdominal aortic aneurysm (AAA), often accompanied by a high mortality rate (1). The occurrence rate of ruptured abdominal aortic aneurysm (rAAA) ranges from approximately 0.01% to 0.21%, with mortality rates as high as 50%–80% (2, 3). At present, the primary therapeutic approaches for rAAA comprise open surgical repair (OSR) and endovascular repair of abdominal aortic aneurysm (EVAR). Over the last decade, continuous technological advancements and device updates have led to a deeper comprehension of EVAR among medical practitioners (4). The utilization rate of EVAR in elective abdominal aortic aneurysm repair has increased from 54% in 2009 to 68% in 2017, surpassing OSR (5). In the setting of emergency rAAA, EVAR is more frequently chosen. Several studies have reported a noteworthy 20%–30% reduction in mortality when compared to OSR (6).

Given the extensive utilization of EVAR in managing rAAA, the choice of endovascular stents, as a critical determinant, can significantly impact surgical procedures and prognostic outcomes (7, 8). Presently, a variety of endovascular stents with distinct characteristics and delivery platforms are available for EVAR implementation. In China, EVAR constitutes a considerable portion of rAAA treatments, yet research comparing diverse endovascular stent therapies for rAAA remains limited. Therefore, leveraging data from a single medical center, this study endeavors to investigate the effects of different endovascular stent types on perioperative outcomes for rAAA.

## 2 Methods

### 2.1 Inclusion and exclusion criteria

In this study, we conducted a retrospective review of patients who underwent EVAR for ruptured abdominal aortic aneurysms at Qingdao University Affiliated Hospital during the period from June 31, 2019, to June 31, 2022. We excluded patients who required simultaneous stent implantation in the renal artery or visceral artery, as well as those with ruptured aneurysms located para-renal, supra-renal, or in the thoracoabdominal region. Patients with isolated iliac artery aneurysms were also excluded from the analysis. Additionally, we excluded patients who underwent open surgery or conversion to open repair during the initial procedure. This study has obtained approval from the Ethics Committee of Qingdao University Affiliated Hospital.

### 2.2 Patients characteristics

Statistical analysis was performed on the demographic variables of all participants, encompassing gender, age, smoking history, alcohol consumption history, and specific laboratory parameters. The laboratory test results were derived from standardized procedures conducted in the Clinical Laboratory of Qingdao University Affiliated Hospital. Furthermore, we conducted statistical analysis on pertinent comorbidities, such as hypertension, diabetes, coronary artery atherosclerotic heart disease, chronic obstructive pulmonary disease (COPD), renal insufficiency, hyperlipidemia, and history of stroke.

### 2.3 Stent selection

Bound by institutional protocols and the exigencies of addressing rAAA within a critical surgical timeframe, the primary stent options consistently employed during the study period were the Gore C3 Excluder (Gore, Flastaff, USA) and the Microport Hercules (MicroPort, Shanghai, China). In cases where a limited number of patients necessitated longer surgical preparation times, there arose a contingent need for the temporary acquisition of alternative stent types. However, the paucity of such instances precluded achieving statistical significance. Consequently, this study's central focus revolves around the comparative analysis of these two stent alternatives. Concerning proximal anchoring techniques, Hercules demonstrates a configuration characterized by renal artery fixation points above, with the fabric component originating beneath the bare stent. The stent's anchoring mechanism relies on the uncoated region situated above the renal arteries, while the sealing function is carried out by the fabric located below this region. In contrast, the Excluder lacks a bare section, and both fixation and sealing are executed from the fabric portion. Furthermore, additional fixation barbs are positioned on the fabric segment, approximately 3–5 mm from the proximal extremity. The selection of the stent type during the surgical procedure is determined by various factors, with the most critical being preoperative imaging examinations such as CTA and DSA, along with the reconstructed data of rAAA. By assessing features like aneurysm diameter and neck length, we choose the most suitable stent from the inventory available at our medical center. Additionally, the decision-making process involves considerations of the patient's financial status and preferences for stents from different sources, such as the higher cost of the Excluder compared to the Hercules.

### 2.4 Procedural details

All patients in this study received treatment under the supervision of the same medical team, with the operating surgeon taking charge of treatment decisions and personally conducting equipment planning measurements and selection. The operating surgeon of this team is a highly experienced vascular surgeon with nearly two decades of experience and an annual caseload of over 100 EVAR procedures. Specialized image analysts within the medical team processed all Computed Tomography Angiography or Digital Subtraction Angiography results using the dedicated Aquarius iNtuition Viewer imaging workstation. The analysis of ruptured abdominal aortic aneurysm morphology included the measurement of the maximum sac diameter, aneurysm neck diameter, neck length, neck angle, and the presence of calcification or thrombosis in the proximal neck. Additionally, statistical analysis was performed on distal anchoring zone characteristics in abdominal aortic aneurysm, such as iliac artery diameter and iliac artery angulation. For intraoperative events, we monitored parameters such as hemodynamic stability, contrast agent volume, aortic balloon occlusion duration, intraoperative blood transfusions or vasopressor administration, mean arterial pressure, and surgical duration. Our analysis focused on endovascular stent devices that were still commercially available and actively utilized. These devices typically possess bifurcated, modular, and fully supported designs, and many of them underwent multiple iterations of graft structures and delivery system design features during their study period.

### 2.5 Outcomes

The primary study outcome was the perioperative mortality rate. Additional study outcomes comprised perioperative complications, reoperation rates, and length of hospital stay. Perioperative complications encompass a range of conditions, including endoleak, graft migration, graft occlusion, and infection. In the event of immediate complications such as endoleak identified in the operating room, intraoperative resolution should be promptly implemented.

### 2.6 Statistical analysis

Frequencies were expressed in percentages and continuous variables in means ± standard deviation. We employed independent samples *t*-tests to compare means between groups after checking for normality. For categorical variables, comparisons were made using either chi-square tests or Fisher's exact tests. To assess time-to-event data, Log-rank tests were used for comparing Kaplan–Meier curves. Additionally, logistic regression was employed to identify variables potentially associated with the study endpoint. A *P*-value of ≤ 0.05 indicated statistical significance in all analyses.

## 3 Results

### 3.1 Patients characteristics

This study enrolled a total of 58 patients who received treatment with two commonly employed endovascular stents in our medical center: Microport Hercules (*n* = 29, 50%) and Gore Excluder (*n* = 29, 50%). Notably, there were no noteworthy dissimilarities in baseline characteristics, encompassing age and gender, between the two treatment cohorts. Nonetheless, it was observed that patients treated with Excluder stents exhibited a notably increased likelihood of concurrent coronary artery disease (13.8% for Hercules vs. 44.8% for Excluder, *P* = 0.009) and a higher proportion of patients with elevated creatinine levels exceeding the normal range (10.3% for Hercules vs. 37.3% for Excluder, *P* = 0.014). No other significant variations in comorbidities were detected between the compared groups (Table 1).

**Table 1:** The characteristics of patients with ruptured abdominal aortic aneurysms (rAAA) undergoing endovascular aneurysm repair (EVAR) at our institution.

Patients characteristics		Hercules *n* = 29 (50%)	Excluder *n* = 29 (50%)	*P*
Demographics				
Male gender		26 (89.7)	23 (79.3)	0.277
Age groups	≤70	21 (72.4)	14 (48.3)	0.060
	>70	8 (27.6)	15 (51.7)	
BMI, M ± SD		23.2 ± 2.6	24.1 ± 2.1	0.412
Comorbidities				
Diabetes mellitus		4 (13.8)	2 (7.9)	0.389
Hypertension		18 (62.1)	22 (75.9)	0.256
Alcohol consumption		12 (41.4)	9 (31.3)	0.412
Smoker		13 (44.8)	14 (48.3)	0.792
Coronary artery atherosclerotic heart disease		4 (13.8)	13 (44.8)	0.009^a^
COPD		1 (3.4)	1 (3.4)	>0.999
Peripheral arterial disease		2 (6.9)	5 (17.2)	0.227
History of stroke		3 (10.3)	2 (6.9)	0.640
Laboratory parameters				
White blood cell count (/L)	≤10 × 10^9^	6 (20.7)	11 (37.9)	0.149
	>10 × 10^9^	23 (79.3)	18 (62.1)	
Platelet count (/L)	≤100 × 10^9^	11 (37.9)	7 (24.1)	0.256
	>100 × 10^9^	18 (62.1)	22 (75.9)	
Hemoglobin (g/L)	Normal/mild anemia	14 (48.3)	17 (58.6)	0.430
	Moderate/severe/very severe anemia	15 (51.7)	12 (41.4)	
Blood creatinine (/L)	≤133	26 (89.7)	18 (62.1)	0.014^a^
	>133	3 (10.3)	11 (37.9)	

BMI, body mass index; COPD, chronic obstructive pulmonary disease.

^a^ Significant difference.

### 3.2 Procedural details

In individuals undergoing treatment with two distinct types of stents, a substantial correlation was noted between the stent variant and specific aneurysm characteristics, as well as events occurring during the procedure. More precisely, patients receiving Excluder stents exhibited significantly shorter aneurysm necks compared to their counterparts treated with Hercules stents (*p* < 0.001). Nonetheless, when considering other attributes related to abdominal aortic aneurysms, such as aneurysm diameter, renal artery angle, calcification or thrombosis in the proximal neck, and characteristics of the distal anchoring zone, no substantial variances were discerned between the two groups. Furthermore, we also examined preoperative hemodynamics and intraoperative events such as surgical duration, aortic balloon occlusion time, contrast agent dosage, and intraoperative mean arterial pressure, but none of them exhibited a significant association with the stent type (Table 2).

**Table 2:** The intraoperative assessments of patients with ruptured abdominal aortic aneurysms (rAAA) undergoing endovascular aneurysm repair (EVAR) at our institution.

Intraoperative assessments		Hercules *n* = 29 (50%)	Excluder *n* = 29 (50%)	*P*
Aneurysm characteristics				
Diameter (mm)		59.9 ± 27.0	67.1 ± 26.3	0.306
Neck length (mm)		39.9 ± 24.1	37.5 ± 20.9	<0.001^a^
Neck diameter (mm)		26.0 ± 7.9	29.1 ± 8.7	0.152
Proximal neck calcification	None	8 (27.6)	5 (17.2)	0.634
	Scattered	19 (65.5)	22 (75.9)	
	More than 2/3	2 (6.9)	2 (6.9)	
Proximal neck thrombosis	None	4 (13.8)	0	0.106
	Scattered	21 (72.4)	23 (79.3)	
	More than 2/3	4 (13.8)	6 (20.7)	
Distal landing zone				
Iliac artery diameter (mm)		16.8 ± 3.5	17.6 ± 2.9	0.342
Iliac artery angulation (^°^)		33.5 ± 5.8	36.2 ± 5.9	0.079
Preoperative hemodynamic instability		14 (48.3)	15 (51.7)	0.790
Intraoperative events				
Contrast volume (ml)		99.8 ± 13.8	104.9 ± 10.8	0.159
Balloon occlusion time (min)		57.0 ± 11.3	62.1 ± 11.0	0.087
Intraoperative blood transfusion		6 (20.7)	8 (27.6)	0.539
Mean arterial pressure (mmHg)		69.9 ± 15.0	72.1 ± 14.1	0.576
Use of vasopressor agents		14 (48.3)	9 (31.0)	0.180
Intraoperative complications		5 (17.2)	9 (31.0)	0.220
Total operation time, M ± SEM (min)		153.8 ± 20.4	165.7 ± 25.5	0.055

^a^ Significant difference.

### 3.3 Outcomes

In the analysis of 2 distinct endovascular stent treatment groups, a statistically significant discrepancy in overall mortality rates was evident (Hercules 27.6%, Excluder 6.9%, *P* = 0.037). (Table 3) Further *post hoc* analysis of mortality rates revealed a notable difference between Hercules and Excluder stents, with lower mortality rates observed in patients treated with Excluder stents, particularly in subgroups of non-alcohol users (*p* = 0.028), non-diabetics (*p* = 0.027), and patients with scattered thrombi in the proximal neck (*p* = 0.046). Other factors showed no significant impact on mortality rates following the application of the two types of endovascular stents. Additionally, there were no significant differences in perioperative complications or length of hospital stay between the treatment groups (Table 4).

**Table 3:** Perioperative outcomes of patients with ruptured abdominal aortic aneurysms (rAAA) undergoing endovascular aneurysm repair (EVAR) at our institution.

Perioperative outcomes	Hercules *n* = 29 (50%)	Excluder *n* = 29 (50%)	***P***
Complications	10 (34.5)	10 (34.5)	>0.999
Outcomes			
Overall mortality	8 (27.6)	2 (6.9)	0.037^a^
Hospital length of stay mean **±** SEM (days)	13.4 ± 13.3	12.1 ± 7.3	0.677

^a^ Significant difference.

**Table 4:** Overall mortality of patients with ruptured abdominal aortic aneurysms (rAAA) treated with two different types of endovascular aneurysm repair (EVAR) stents at our institution.

Characteristics		Hercules *n* = 21 (43.8%)	Excluder *n* = 27 (56.3%)	***P***
Demographics				
Male gender		19 (90.5)	21 (77.8)	0.242
Age groups	≤70	15 (71.4)	13 (48.1)	0.105
	>70	6 (28.6)	14 (51.9)	
BMI, M ± SD		22.9 ± 2.5	23.3 ± 2.4	0.251
Comorbidities				
Diabetes mellitus		4 (19.0)	0 (0)	0.027^a^
Hypertension		13 (61.9)	21 (77.8)	0.325
Alcohol consumption		12 (57.1)	7 (25.9)	0.028^a^
Smoker		10 (47.6)	13 (48.1)	0.934
Coronary artery atherosclerotic heart disease		4 (19.0)	11 (40.7)	0.108
COPD		1 (4.8)	1 (3.7)	0.856
Peripheral arterial disease		2 (9.5)	5 (17.2)	0.227
History of stroke		2 (9.5)	2 (6.9)	0.792
Laboratory parameters				
White blood cell count	≤10 × 10^9^	6 (28.6)	10 (37.0)	0.537
	>10 × 10^9^	15 (71.4)	17 (63.0)	
Platelet count	≤100 × 10^9^	4 (19.0)	7 (25.9)	0.574
	>100 × 10^9^	17 (81.0)	20 (74.1)	
Hemoglobin	Normal/mild anemia	14 (66.7)	17 (63.0)	0.552
	Moderate/severe/very severe anemia	7 (33.3)	10 (37.0)	
Blood creatinine	≤133	19 (90.5)	18 (66.6)	0.052
	>133	2 (9.5)	9 (33.4)	
Aneurysm characteristics				
Diameter		60.0 ± 28.4	66.4 ± 26.0	0.422
Neck length		39.0 ± 22.7	21.5 ± 12.3	0.001^a^
Neck diameter		26.5 ± 7.9	29.5 ± 8.9	0.230
Proximal neck calcification	None	7 (33.3)	6 (22.2)	0.648
	Scattered	12 (57.1)	17 (63.0)	
	More than 2/3	2 (9.5)	4 (14.8)	
Proximal neck thrombosis	None	4 (13.8)	0	0.046^a^
	Scattered	21 (72.4)	23 (79.3)	
	More than 2/3	4 (13.8)	6 (20.7)	

^a^ Significant difference.

### 3.4 Factors associated with mortality on multivariable regression

Based on the results of univariate analysis and clinical relevance, we selected eight variables for multivariate regression analysis. The study highlighted that the selection of the endograft emerged as a significant independent factor associated with mortality. Specifically, treatment with Hercules was associated with a higher risk of mortality in comparison to Excluder (odds ratio [OR] 2.97, 95% confidence interval [CI] 1.31–6.7). Additionally, perioperative complications during EVAR were significantly linked to an increased risk of mortality (OR 20.70, 95% CI 1.14–376.70). However, no other factors showed a significant correlation with mortality in rAAA according to the findings (Table 5).

**Table 5:** Factors associated with mortality on multivariable regression analysis.

Risk factors	OR [95% CI]
Excluder vs. Hercules	0.06 (0.00–1.31)^a^
Platelet count	0.23 (0.03–2.11)
Intraoperative hemodynamics	9.41 (0.65–136.20)
Intraoperative complications	20.70 (1.14–376.70)^a^
Neck length	1.01 (0.97–1.07)
Contrast volume	0.94 (0.88–1.00)
Balloon occlusion time	1.00 (0.97–1.02)
Total operation time	1.06 (1.00–1.13)

^a^ Significant difference.

## 4 Discussion

RAAA is a highly critical condition with a mortality rate as high as 50% (9, 10). Recently, the use of EVAR for managing rAAA has gained widespread recognition, thanks to technological advancements and updates in endovascular stents. Studies have demonstrated that EVAR can significantly reduce perioperative mortality and 5-year mortality in rAAA cases (4, 11). The choice of endograft is a pivotal factor in the success of EVAR treatment (11). In our study, we analyzed partial clinical outcomes of patients who underwent endovascular repair for rAAA at a single medical center in eastern China during a 3-year period. Our findings revealed that the type of endograft used for EVAR was independently associated with the 30-day mortality rate. Specifically, patients treated with Microport Hercules endograft showed a significantly higher 30-day mortality rate compared to those treated with Gore Excluder endograft. Interestingly, the mortality rate among patients treated with Excluder endograft was only 6.9%, possibly one of the lowest reported in the literature (12–14). Although this association does not imply causation or establish that a specific endograft leads to superior outcomes in rAAA rupture, it is necessary to discuss these findings and explore potential explanations for this observation.

The observed disparity in mortality rates among different endografts is likely influenced by multiple factors, including clinical, anatomical, and operator-related variables (15, 16). However, there are certain specific distinctions worth highlighting among these endografts. The two devices studied in this research exhibit unique characteristics in terms of their structures, delivery systems, and materials. The Gore Excluder endograft, crafted from nitinol-based material, stands out for its ability to achieve immediate sealing within the infrarenal aorta. Its bimodular design suggests a faster deployment speed compared to tri-modular endografts, as it does not require suprarenal fixation, thereby eliminating a step during deployment (17–19). This streamlined deployment process results in reduced operative time, which can have crucial implications for patients with ruptured abdominal aortic aneurysms. Shorter operative time may lead to decreased blood loss, reduced shock duration, and less systemic hypoxia, potentially contributing to a lower risk of mortality. Thus, the Gore Excluder's attributes may render it more versatile and applicable in cases involving high-risk patients with conditions such as coronary artery disease, renal insufficiency, or shorter aortic neck lengths.

Remarkably, our findings revealed a noteworthy association between the utilization of Excluder endografts and potentially reduced mortality rates in specific patient subgroups, including those without diabetes, non-alcohol users, and patients with scattered thrombi in the proximal neck. Prior studies have suggested potential protective effects of diabetes on the aortic wall, with lower total mortality rates observed after acute AAA repair in patients with type 2 diabetes compared to those without diabetes (20, 21). This trend has been supported by statistical data from various countries, such as the UK and Sweden (22, 23). However, it is essential to acknowledge that poorly controlled blood glucose has been recognized as a significant risk factor for rAAA in other studies (24). At our medical center, diabetes diagnosis was based on criteria involving random blood glucose and glycated hemoglobin levels surpassing abnormal values. Consequently, patients without diabetes might experience less impact from abnormal blood glucose levels, thus potentially contributing to their lower mortality rates (25). Similarly, regarding the correlation between alcohol consumption and AAA, although there is ongoing debate, an increasing body of evidence substantiates its potential to accelerate the onset and even rupture of AAAs. The primary mechanism is likely associated with alcohol-induced upregulation of matrix metalloproteinase expression (26). The observation of lower mortality rates in patients without a history of alcohol consumption could be linked to their comparatively better vascular conditions and the surrounding vascular environment. In the context of AAA, the presence of proximal neck thrombosis is considered one of the risk factors for rupture and also predisposes patients to post-EVAR thrombosis due to potential embolization (27, 28). Among the rAAA patients included in this study, a substantial portion (84.5%) had thrombosis in the proximal neck, with a considerable majority (91.8%) of these cases having thrombi exceeding half of the circumference. The presence of scattered thrombi in the proximal neck might contribute to simplified surgical procedures and reduced rates of postoperative complications. Taken together, in situations where aortic conditions are relatively favorable, the use of Excluder endografts may play a role in contributing to reduced mortality rates to some extent.

In our analysis of risk factors for mortality in patients with rAAA, we observed that perioperative complications were significantly associated with higher mortality rates. In previous studies, common complications during EVAR typically encompass endoleaks, stent delivery challenges, insufficient distal expansion, arterial rupture, and distal thrombosis or embolism, among others (29, 30). In our study group, 14 patients met our specific criteria for intraoperative complications, all classified as Type I or Type III endoleaks. We didn't categorize Type II endoleaks found during the procedure as complications, as they don't represent genuine intraoperative failures and can be effectively managed with diligent follow-up. The occurrence of intraoperative endoleaks appeared to be linked to issues with stent specifications, deployment, or fixation, and the incidence was notably higher than that observed in standard EVAR (31, 32). Patients with RAAA face a critical and rapidly evolving condition, and conducting a preoperative CTA examination could potentially delay life-saving interventions. Furthermore, complex factors such as the anatomy of the aneurysm neck length can influence the occurrence of intraoperative endoleaks, which are more prevalent in RAAA cases compared to standard EVAR procedures. Within the scope of our study comparing two different stent types, we found no significant difference in the occurrence of endoleaks. Similarly, given the urgency of RAAA cases, there were no noteworthy distinctions in the prevention and management of endoleaks between the two stent types. In terms of treatment, the standard approach involves intraoperative balloon angioplasty or extending stent placement for control, with confirmation of endoleak resolution through DSA (33). Preventive measures primarily focus on ensuring patient safety while optimizing imaging studies within the limited timeframe. This includes choosing appropriately sized stents based on imaging findings and maintaining a high level of alertness and vigilance during the surgical procedure. Immediate corrective actions are taken upon detecting Type I or Type III endoleaks.

Regarding other inter-group differences in indicators such as length of hospital stay, hemodynamics, and platelet levels, we did not find them to be independent risk factors for mortality in rAAA patients. Although some animal experiments have suggested that the use of platelet inhibitors might reduce the mortality rate of AAA, this conclusion is not widely accepted in clinical practice due to the different mechanisms of abdominal aortic aneurysm formation and rupture between mouse models and humans (34–36). Our findings also support the notion that platelet levels may not have a significant impact on mortality in rAAA patients undergoing EVAR.

This research has its set of constraints. To begin with, it's a retrospective investigation carried out at a single center, which has resulted in a relatively modest cohort, diminishing the strength of our findings in contrast to prospective studies. Furthermore, due to specific policies within this center, there weren't any substantial statistical disparities for the less commonly employed stent types. Moreover, this study exclusively concentrated on the initial repercussions of EVAR, without delving into the analysis of its intermediate to long-term consequences. Therefore, additional research is imperative to provide more substantial support for our conclusions.

## 5 Conclusion

In this study, our preliminary experience showed that the type of endograft used and perioperative complications could potentially serve as independent risk factors that influence mortality rates. The observed significant differences in outcomes among the various endografts highlight the need for further investigation in this area.

## Data Availability

The original contributions presented in the study are included in the article/Supplementary Material, further inquiries can be directed to the corresponding authors.
